# Evaluation of the *Young*, *Deadly*, *Syphilis Free* multi-media campaign in remote Australia

**DOI:** 10.1371/journal.pone.0273658

**Published:** 2022-09-09

**Authors:** Belinda D’Costa, Roanna Lobo, Amanda Sibosado, Justine E. Leavy, Gemma Crawford, James Ward

**Affiliations:** 1 Sexual Health and Blood-Borne Virus Applied Research and Evaluation Network, Collaboration for Evidence, Research and Impact in Public Health, School of Population Health, Faculty of Health Sciences, Curtin University, Perth, Western Australia, Australia; 2 Curtin School of Allied Health, Faculty of Health Sciences, Curtin University, Perth, Western Australia, Australia; 3 Collaboration for Evidence, Research and Impact in Public Health, School of Population Health, Faculty of Health Sciences, Curtin University, Perth, Western Australia, Australia; 4 South Australian Health and Medical Research Institute, Adelaide, South Australia, Australia; 5 UQ Poche Centre for Indigenous Health, School of Public Health, University of Queensland, Brisbane, Queensland, Australia; University of South Australia, AUSTRALIA

## Abstract

**Introduction:**

Since 2011, remote Australian Aboriginal and Torres Strait Islander communities have experienced an outbreak of infectious syphilis, with young people aged 15–29 years over-represented in notifications. The *Young Deadly Syphilis Free* multi-media campaign was implemented in 12 remote regions in four Australian jurisdictions over nine months from 2017–2018. Campaign components included television and radio advertisements, social media posts, and health promotion resources available via a dedicated website. The aim of this research was to evaluate the impacts (proximal, mediator, distal) of the *Young Deadly Syphilis Free* campaign for young Aboriginal people and health and community workers residing in remote campaign regions.

**Methods:**

A cross-sectional (post-only) evaluation design was used. Data were collected through online surveys; metrics for social media (Facebook, Instagram) were also collected to determine campaign engagement via social media. A 22-item *young people survey* assessed campaign awareness, exposure, message recognition and diagnostics (proximal variables); along with intended behaviour and knowledge and attitudes (mediator variables). A 24-item *health and community worker survey* assessed campaign awareness, exposure, message recognition and diagnostics (proximal variables); and changes in professional practice (distal variable). Descriptive statistics summarised demographic characteristics and univariate analysis examined associations between key variables.

**Results:**

Just over half (*n* = 25, 58%) of young people and three quarters (*n* = 36; 75%) of health and community workers were aware of the campaign. Recognition of key campaign messages was high for both participant groups (>64%), and television, Facebook, and website were the most common campaign exposure routes. Positive impacts on intended behaviour (young people) and professional practice (health and community workers) were also reported. Facebook was effective in engaging some young people in campaign content and was preferred by young people for accessing information.

**Conclusion:**

The findings point to the value of utilising a multi-media campaign in raising awareness about syphilis among young Aboriginal people and health and community workers in remote Australian regions. A longer-term campaign that accommodates the diverse needs of Aboriginal young people from geographically remote communities would optimise campaign impacts and support behavioural change.

## Introduction

Since 2011, northern and central southern Australia has experienced a syphilis outbreak, primarily affecting young Aboriginal and Torres Strait Islander people (hereafter respectfully referred to as Aboriginal people) living in regional and remote communities [1]. Syphilis is highly infectious and easily transmitted, sexually or from mother to child. Aboriginal people are disproportionately affected [1]. The 2020 notification rate for the Aboriginal population was 101.5 per 100,000 –a 241% increase from 2011 and more than five times the rate for the non-Aboriginal population (18.1 per 100,000) [2]. Between 2011 and 2020, there were almost 4,000 syphilis notifications reported among Aboriginal people residing in outbreak regions in Queensland (Qld), the Northern Territory (NT), Western Australia (WA), and South Australia (SA) [3]. More than half occurred among young Aboriginal people aged 15–34 years.

The *Young Deadly Syphilis Free* (*YDSF*) campaign was piloted in response to the outbreak. The campaign aimed to increase awareness of the current syphilis outbreak and promote testing for syphilis and other sexually transmissible infections (STIs) across 12 outbreak regions in Qld, WA, SA, and the NT. *YDSF* was the first multi-media, multi-jurisdictional sexual health campaign implemented in remote Australian Aboriginal communities. Two campaign audience groups were identified: 1) primary—young Aboriginal people aged 15–34 years; 2) secondary—health and community workers (HCW) residing in remote regions. The campaign comprised television (TV) and radio advertisements (ads), social media, and a suite of resources hosted on the *Young Deadly Free* (*YDF*) website. The *YDSF* rollout over nine months from 2017–2018 marked the first time ads about such a sensitive topic (STIs) had been shown on TV in remote communities to a mixed audience. Previously sexual health promotion typically adopted a more targeted approach and involved separate discussions with women and men per cultural protocols regarding women’s business and men’s business.

This paper presents cross-sectional evaluation survey findings from the *YDSF* campaign. The independent evaluation sought to assess the impacts (proximal, mediator, distal) of the campaign for young Aboriginal people aged 15–34 years and health and community workers in the campaign regions. In accordance with the work of Bauman, Smith, Maibach, and Reger-Nash [4], the proximal variables included campaign awareness, exposure, message recognition, and diagnostics for both participant groups. For young people (YP), mediator impact variables were intended behaviour, knowledge, and attitudes. The influence of the campaign on health and community workers’ professional practice measured the distal impact of the campaign. Engagement with the campaign via social media was also assessed.

### Campaign overview

The South Australian Health and Medical Research Institute (SAHMRI) developed the campaign, informed by formative research undertaken by the Aboriginal Nations Torres Strait Islander HIV Youth Mob (ANTHYM) in seven sites across Qld, WA, and the NT. The formative research consulted with YP aged 15–29 years (*n* = 82; 44 males and 38 females) on the campaign title, *Young Deadly Syphilis Free*, the contents of the TV ads, the messages that would encourage syphilis testing among young people, and media preferences. The outcomes of the formative research determined the campaign components and delivery channels, which are summarised in Table 1.

**Table 1:** *Young Deadly Syphilis Free* campaign components and delivery.

Components	Activity	Time period
Television	TV ads (*n* = 2)	1 July 2017–31 March 2018
	• Paid spots^1^ (*n* = 1,280)	
	• Bonus and filler spots (*n* = 1,487)	
Radio	Radio ads (*n* = 4)	1 July 2017–31 March 2018
	• Paid spots (*n* = 668)	
	• Bonus and filler spots (*n* = 120)	
Facebook	Facebook posts (*n* = 324)	1 July 2017–31 March 2018
	Facebook ads (*n* = 9)	1 July 2017–31 March 2018
Instagram	Instagram posts (*n* = 262)	1 July 2017–31 March 2018
	Instagram ads (*n* = 9)	1 July 2017–31 March 2018
Divas Chat^2^	*YDSF* advertisement banners (*n* = 2)	1 July 2017–30 Nov 2017 and
		1 March 2018–31 March 2018
*Young Deadly Free* website	Syphilis factsheets (*n* = 2)	1 July 2017–31 March 2018
	Syphilis posters (*n* = 2)	
	Syphilis infographics (*n* = 9)	
	Syphilis animation (*n* = 1)	

^1^ Data provided for TV and radio were for spots, which refers to advertising space typically either 30 or 60 seconds in length. The ad spots run at specified times during specified programs and vary considerably in cost.

^2^ Divas Chat is a free social networking service popular among young Aboriginal people in remote communities for it only requires a 3G network coverage.

Two 30-second TV ads (see TV ad 1 and TV ad 2), were produced and filmed in SA, featuring paid local Aboriginal actors. Ads were screened across remote station networks in WA, SA, Qld, and the NT and promoted the importance of syphilis testing. Taglines included *I’ve had my syphilis test*, *have you had your*s? and *Get tested today*.

Supporting the TV ads were 16 radio ads broadcast across major remote radio station networks. Radio stations were provided with four scripts to adapt to the local context and ensure adherence to cultural protocols. Taglines included *I’ve had mine*, *have you had yours*? and *Get tested at the clinic today*. Radio stations produced ads using local Aboriginal voiceover artists or language speakers to ensure cultural appropriateness. TV and radio advertising spend across the nine months was approximately $105,000 (AUD), with TV advertising accounting for 75% of the budget. TV and radio stations provided bonus and filler spots at no charge, with an estimated value of approximately $68,000 (AUD).

SAHMRI developed *YDSF* campaign resources in consultation with remote communities and health practitioners across the regions (e.g., posters, infographics, factsheets, and animations). Resources were housed on the *YDF* website and disseminated via USBs to health services in affected regions with limited internet connectivity.

The social media component used Divas Chat, Facebook, and Instagram to promote *YDSF* awareness via posts and paid ads. Posts primarily consisted of TV ads (videos and stills) and resources. To enhance content visibility, ads (*n* = 9) were purchased (costing $300 (AUD)). Paid advertising promoted TV ads, resources, and directed traffic to the webpage.

## Methods

### Design

SAHMRI commissioned the WA Sexual Health and Blood-borne Virus Applied Research and Evaluation Network (SiREN) to conduct an independent evaluation of the *YDSF* campaign. The evaluation used cross-sectional (post-only) online surveys with the primary and secondary target groups (*YP survey* and *HCW survey* respectively); social media metrics were also collected. The Aboriginal Health Council of South Australia’s Aboriginal Health Research Ethics Committee (04-17-722) and the Curtin University Human Research Ethics Committee (HRE2017-0605) provided ethical approval. The rationale provided for a low-risk study, which did not require parental consent, was accepted by the Human Research Ethics Committees.

### Participants

We recruited young people through online platforms, including the *YDSF* Facebook and Instagram pages, ads on Facebook and Divas Chat, and pop-up boxes on the *YDF* website. Participants in the *YP survey* identified as Aboriginal and/or Torres Strait Islander, were aged between 15–34 years, and residents of WA, SA, Qld, or the NT. Four one-week duration paid Facebook ads generated survey awareness among the campaign’s target audience. Three of the four ads targeted males and females aged 18–35 years. The fourth ad specifically targeted males aged 15–35 years to encourage greater representation from Aboriginal males in the *YP survey*. We offered an iPad via a prize draw to young people as an incentive for completing the survey. HCWs were eligible to participate in the *HCW survey* if they worked in WA, SA, Qld, or the NT. Recruitment occurred via the *YDSF* newsletter, whereby subscribers (*n* = 350) received a survey link via direct email.

### Survey measures

Surveys were self-completed online using Qualtrics™ software, averaging five minutes to complete. The 22-item *YP survey* collected data on campaign awareness, exposure, message recognition and diagnostics, media consumption, social media behaviour and engagement, and changes in knowledge, attitudes, and behavioural intentions concerning syphilis and testing. The 24-item *HCW survey* assessed campaign awareness, exposure, message recognition and diagnostics, social media behaviour and engagement, and changes in professional practice. Surveys included previously trialled questions [5, 6] using forced response, multiple-choice, three-point scales (e.g., yes/no/don’t know), and a five-point Likert scale (e.g., where 1 denoted ‘disagree’ and 5 denoted ‘agree’). The campaign team tested survey logic before going live, resulting in minor modifications. Surveys remained open for five and a half weeks.

A combination of prompted recall and prompted recognition assessed campaign awareness [7, 8]. An open-ended question assessed prompted recall whereby participants indicated if they had seen or heard any recent syphilis ads. Responses were coded as ‘yes’ when the participant was able to either correctly describe details of one or both of the TV ads (e.g., *girl has syphilis and loses baby*), or correctly refer to the campaign title or taglines (e.g., *Young Deadly Syphilis Free*; *Get tested today*). Prompted recognition required participants to recognise the TV ads based on the images provided (‘yes’ or ‘no’). Campaign awareness was calculated as the total number of individuals who either recalled the advertisement or recognised it through prompting, that is, total awareness = prompted recall + prompted recognition.

Exposure was determined by asking where participants had heard about, or seen, the campaign. Options included: television; Facebook; Instagram; Divas Chat; Twitter; radio; *Young Deadly Free* website; other.

Seven statements assessed recognition [5]. Participants indicated key campaign messages they recalled, for example, *Syphilis can harm you and the people you sleep with too* and *Young people should use condoms and get tested for syphilis*.

Perceived message effectiveness of TV ads was assessed using diagnostics related to believability, relevance, peer-sharing/client sharing, newness, ease of understanding, enjoyment, and appeal. Participants indicated agreement/disagreement with specific statements; for example, *I enjoyed watching these ads* [6].

Questions about syphilis, prevention, stigma and shame, testing, and contraception use assessed young people’s self-reported changes in knowledge, attitudes, and behavioural intentions. To determine campaign effects on professional practice, HCWs were asked if, and how, their engagement with young people had changed concerning syphilis and testing.

Media consumption was assessed by asking young people to indicate the length of time spent reading, listening to, or using: Facebook; Instagram; Twitter; Divas Chat; radio; television; or other media.

### Social media measures

Facebook and Instagram Insights provided data on social media engagement during the campaign period. *YDSF* Facebook data included: page likes (when an individual ‘likes’ a page, indicating they want to see content from it); follower profile (demographic information about the people who follow the page); number of news feeds reached by posts (the number of people who had a post from the page enter their news feed); posts receiving greatest engagement (total number of likes, shares, comments, views, link clicks for a post); and total engagement (total number of times all posts were shared, reacted to, commented on, or clicked on).

*YDSF* Instagram data included: number of followers (total number of users who follow the page); demographic profile of followers (demographic information of users who follow the page); number of posts (total number of times content was published on the page); and posts receiving the most likes (users ‘like’ content to indicate appreciation and support of content).

### Analysis

For analysis, Qualtrics survey data were exported to SPSS (version 23) (IBM Corp., Armonk, NY 2017). *YP survey* data (*n* = 61) were cleaned (incomplete surveys and those not meeting eligibility criteria excluded) leaving a final sample of *n* = 43. Similarly, data from the *HCW survey* were cleaned (*n* = 55), resulting in a complete data set of *n* = 48. Descriptive statistics summarised demographic characteristics of all eligible survey participants. Associations between campaign variables were examined by univariate analysis and was limited to data collected from young people and health and community workers who were aware of the campaign.

## Results

### Survey participant demographics

Characteristics of all eligible survey participants are shown in Table 2.

**Table 2:** Characteristics of eligible survey participants.

Demographic characteristic	YP Survey^1^ (*n* = 43)	HCW^1^ Survey (*n* = 48)
	*n* (%)^2^	*n* (%)^2^
**Identify as Aboriginal**		
• Yes	43 (100)	15 (31)
• No	-	32 (67)
**English first language**		
• Yes	39 (91)	46 (96)
• No	3 (7)	1 (2)
**Gender identity**		
• Female	31 (72)	37 (77)
• Male	10 (23)	10 (21)
• Other	1 (2)	-
**Age group (years)**		
• 15–17	13 (30)	-
• 18–24	22 (51)	4 (8)
• 25–34	8 (19)	15 (31)
• 35–44	-	6 (12)
• 45 and older	-	23 (48)
**Highest level of education**		
• Left school before finishing Year 10	10 (23)	1 (2)
• Completed Year 10	-	-
• Completed Year 12	19 (44)	3 (6)
• Completed a diploma or university degree	11 (26)	4 (8)
	2 (5)	N/A
• Completed trade/diploma certificate	N/A	9 (19)
• Completed university /college	N/A	29 (60)
**Primary work**		
• Health Worker	N/A	10 (21)
• Nurse	N/A	15 (31)
• Doctor	N/A	1 (2)
• Community Worker	N/A	6 (12)
• Other	N/A	15 (31)
**State/territory of residence/work**		
• WA	8 (19)	11 (23)
• SA	12 (28)	9 (19)
• NT	11 (26)	15 (31)
• Qld	12 (28)	13 (27)
**Preferred communication**		
• Television	5 (12)	N/A
• Radio	1 (2)	N/A
• Facebook	27 (63)	N/A
• Divas Chat	1 (2)	N/A
• Other	2 (5)	N/A
**Media consumption, 2hrs or more per day** ^**3**^		
TV	14 (32)	N/A
Radio	2 (5)	N/A
Facebook	24 (56)	N/A
Instagram	12 (28)	N/A
Divas Chat	7 (16)	N/A
Twitter	2 (5)	N/A
Other (e.g., SnapChat)	2 (5)	N/A

^1^ Columns may not equal 100% due to percentages being rounded to the nearest whole number and item non-response.

^2^ Number of eligible survey participants has been used as the dominator when calculating the proportions (%).

^3^ Survey participants indicated hours spent per day on all media; column does not sum to 100%.

Almost three-quarters of eligible *YP survey* participants were female (72%, *n* = 31), and a majority reported English as their first language (91%, *n* = 39). Just over half (51%, *n* = 22) were aged 18–24 years. The highest level of education achieved was Year 10 (44%, *n* = 19). Most participants (79%, *n* = 34) rated their health positively.

The majority of eligible *HCW survey* participants were also female (77%, *n* = 37). Almost one-third (31%, *n* = 15) were aged between 25–34 years, while just under half (48%, *n* = 23) were aged between 45–64 years. Nursing (31%, *n* = 15) and Health Worker (21%, *n* = 10) were the most commonly reported professions.

Among eligible *YP survey* participants, Facebook was the most utilised communication platform. Most young people (56%, *n* = 24) spent two or more hours per day on Facebook. TV viewing was also high among young people, with almost one in three reporting two hours or more per day watching television. Divas Chat, Twitter, and radio were less likely to be utilised.

The following findings pertain to participants who were aware of the campaign.

### Campaign awareness

Of *YP survey* participants, 12% (*n* = 5) could correctly recall the campaign (prompted recall). When prompted with images, just under half (47%, *n* = 20) recognised the campaign (prompted recognition); total campaign awareness was 58% (*n* = 25). Among *HCW survey* participants, total campaign awareness was higher (75%, *n* = 36). Almost one third (31%, *n* = 15) could correctly recall the campaign (prompted recall); when prompted with images, 44% (*n* = 21) recognised the campaign.

### Campaign exposure

TV, Facebook, and the *YDF* website were common campaign exposure channels. *YP survey* participants typically reported exposure via TV (60%, *n* = 15); almost one-third reported seeing the campaign on Facebook (32%, *n* = 8). *HCW survey* participants were primarily exposed via TV and the *YDF* website (56%, *n =* 20 respectively); more than one-quarter (28%, *n* = 10) saw the campaign on Facebook. Exposure via radio, Divas Chat, and Instagram was lower, ranging from 0% to 8% (*n* = 3) across both groups.

### Recognition of campaign messages

Key message recognition was high among participants to both surveys. Three quarters (76%, *n* = 19) of *YP survey* participants identified four of the key messages: *syphilis is an infection that is spreading through our communities*; *you can get syphilis if you have sex without a condom*; *syphilis can harm you and the people you sleep with too*, and *syphilis can harm unborn babies*. The other two campaign messages, *young people should use condoms and get tested for syphilis* (68%, *n* = 17), and *pregnant women should get tested for syphilis* (64%, *n* = 16), were identified by around one-third of participants. Message recognition was higher among *HCW survey* participants, ranging from 83% for *you can get syphilis if you have sex without a condom* (*n* = 30) to 94% for *syphilis is an infection that is spreading through our communities* (*n* = 34).

### Campaign diagnostics

Table 3 presents responses to the TV ads by *YP survey* participants. The majority agreed the TV ads grabbed their attention and were easy to understand; one-third agreed ads were relevant to them. The majority of *HCW survey* participants agreed the TV ads were believable and relevant to young people in their community (Table 3).

**Table 3:** Young people and HCW responses to TV ads.

Response to TV ads (agreement)	YP	HCWs
	% (*n*)	% (*n*)
TV ads grabbed my attention	80% (*n* = 20)	N/A
TV ads were easy to understand	80% (*n* = 20)	89% (*n* = 32)
TV ads were believable	72% (*n* = 18)	97% (*n* = 35)
TV ads told me something new	68% (*n* = 17)	N/A
I would talk about TV ads with my friends	60% (*n* = 15)	N/A
I would talk about the TV ads with young people in my community	N/A	92% (*n* = 33)
I enjoyed watching TV ads	56% (*n* = 14)	N/A
TV ads would appeal to young people in my community	N/A	81% (*n* = 29)
TV ads were relevant to me	36% (*n* = 9)	N/A
TV ads were relevant to young people in my community	N/A	97% (*n* = 35)

### Impact on primary target group’s intended behaviour, knowledge, and attitudes

More than half (60%, *n* = 15) of *YP survey* participants reported that TV ads influenced intentions to change their behaviour. Commonly reported intended change was *use condoms when having sex* (52%, *n* = 13), followed by *get tested for syphilis* (44%, *n* = 11). Ads were less likely to encourage greater dialogue among young people about syphilis. Around one-third (36.0%, *n* = 9) agreed that ads encouraged them to *talk to family and friends about the importance of getting tested for syphilis*.

Just over half (52%, *n* = 13) believed ads *increased knowledge about syphilis in the community*. Around one-third (36%, *n* = 9) agreed ads *helped people in the community understand how to stay syphilis free;* a slightly smaller proportion (32%, *n* = 8) agreed that ads *changed the way people in the community think about syphilis*.

Less than one-third (28%, *n* = 7) agreed that the ads helped *reduce the stigma and shame about syphilis*. One-quarter agreed the ads *helped to reduce the shame and stigma about getting tested for syphili*s (24%, *n* = 6) and that *young people felt more confident to get tested regularly for syphilis* (24%, *n* = 6).

### Impact of television campaign on secondary target group’s professional practice

Most *HCW survey* participants (89%, *n* = 32) reported the campaign positively influenced professional practice. The most common change made by participants was to *talk more often to young people in the community about STIs and the importance of testing* (64%, *n* = 23).

After seeing the ads, 44% (*n* = 16) said they *talk more often to young people attending their health service about STIs and the importance of testing*, and 42% (*n* = 15) claimed they *offer more opportunistic testing to young people* and *free condoms to all young people who attend their health service*. Of those who indicated the campaign had affected their professional practice in other ways (36%, *n* = 13), participants commonly reported the ads provided a non-confrontational way to broach the topic of STI testing with young people.

### Campaign engagement via social media

Four hundred and fifty people liked the Facebook page during the campaign period, predominantly females (70%); around one-third aged 25 to 34 years (31%). Engagement with the 324 published Facebook posts was greatest for the two TV ads (935 and 364 likes). Content from the Facebook page reached 139,870 news feeds. Total engagement for published posts on the Facebook page was 12,653. The Instagram account had 106 followers at the end of the campaign period, predominantly females (67%); just under half aged 25–34 years (45%). During the campaign period, 262 Instagram posts were published. Screenshots of the syphilis animation and one TV ad received the most likes (19 likes, respectively).

## Discussion

Awareness of the *Young Deadly Syphilis Free (YDSF)* campaign was almost 60% among young people and 75% among HCWs. TV, Facebook, and website were the most common campaign exposure routes for all participants. More than half of the participants reported that the TV ads yielded a positive impact on their behavioural intentions, with young people encouraged to use condoms while HCWs were motivated to talk more often to young people about STIs and the importance of testing. The ads, however, had little impact on shifting negative STI attitudes or reducing the stigma associated with syphilis. Similarly, despite formative research, the campaign had minimal effect in encouraging conversations among young people about sexual health and the importance of STI testing. Facebook was a useful engagement strategy, particularly for females and those aged 25–34 years. With a relatively modest investment, results indicate audience engagement with the campaign.

### Campaign awareness, exposure, and message recognition

*YDSF* post-campaign awareness levels were consistent with mass media campaigns for health issues in general [6]. More than half were exposed to the campaign via TV and almost one third via Facebook. Most HCWs (83%-94%), and more than three out of five (68%-76%) young people, recognised key campaign messages. The absence of comparable campaigns in the literature, specifically those of similar scale, strategic approach, and target audience limits the interpretation of these findings. Cautious comparisons may be made with a NT media campaign to encourage STI testing among urban young people aged 15–29 that reported 70% of the young people had seen TV ads and identification of campaign messages ranged from 32%-70% [9]. Another campaign targeting young people in the Torres Strait reported 85% had heard of a 12-episode educational radio drama about family, love, and relationships; less than half (47%) could recall storylines or characters [10]. Significantly for both campaigns, awareness was limited to a single yes/no question, which may signal validity concerns for findings from this question [11].

### Campaign diagnostics

Participants reported *YDSF* TV ads were believable, attention-grabbing, and easy to understand. Findings underscore the value of community consultation and formative research in campaign development. Evaluation findings from earlier sexual health campaigns targeting Aboriginal young people, such as *Snake Condoms* and *Kasa Por Yarn*, highlight the benefit of local community involvement in campaign development. The community has a critical role in facilitating not only the cultural relevance of a campaign but ultimately its success [10, 12, 13].

Most *HCW survey* participants perceived ads were relevant to young people. In contrast, one-third of *YP survey* participants believed the ads were personally relevant. It is unclear whether this inconsistency between the primary and secondary target group may result from location, social norms, topic stigmatisation, or other factors related to perceived message effectiveness. A previous evaluation of bloodborne virus and STI campaigns in WA targeting young Aboriginal people reported similar findings. While participants trusted the information and reported the campaigns were easy to understand, less than half believed the campaigns were personally relevant [14]. Such findings reinforce the importance of formative testing to determine the appropriateness of campaign materials while also highlighting the challenges of developing ads with universal appeal across a non-homogenous group of individuals. The findings also suggest a possible misalignment between young people’s risk perception and participation in risk practices. Previous research has similarly reported dissonance between perceived and actual engagement in risk practices among Aboriginal young people [15]. Improved understanding of the domains of perceived message effectiveness in the context of Aboriginal sexual health promotion campaigns is needed. Further investigation of the role peers play in influencing risk perception is warranted.

### Campaign impacts on target groups

A high proportion of young people reported the TV ads taught them something new, and just over half believed the ads increased knowledge about syphilis in the community. However, knowledge on syphilis prevention remained limited post-campaign. This finding may suggest the need for a greater focus on prevention behaviours in future campaign iterations, or it may be symptomatic of the campaign’s short timeframe and the resultant limitations on the ability to observe any significant effects [16].

TV ads had a negligible impact on shifting negative STI attitudes and encouraging greater dialogue among young people about sexual health and the importance of STI testing. The sensitivity and shame surrounding STIs and sexual health for Aboriginal people is commonly reported in the literature and may account for this finding [17–20]. However, negative attitudes towards discussing STIs and sexual health are not culturally bound, with reports of STI related stigma or shame also prevalent among non-Aboriginal young people. Such findings support further action to change the dominant sexual health discourse for all young people [21–23].

TV ads positively impacted young people’s intentions to use condoms when having sex and get tested for syphilis. Behavioural intention is an important predictor of behavioural change [24]. However, for a campaign to have a lasting behavioural impact, sustained efforts are required, particularly for young people heavily influenced by social norms [25]. Such measures must consider the range of complexities that influence STI prevention and testing behaviours of young Aboriginal people, which in addition to attitudinal and social influences, can include health service influences [26, 27].

TV ads also had a promising impact on HCWs professional practice, with workers encouraged to engage in conversations with young people in the community about STIs and the importance of testing. This suggests the important role of the TV ads as conversational tools, supporting the initiation of discussions of a sensitive nature between health professionals and young people. This underscores the value of health resources for health professionals.

### Campaign engagement via social media

Facebook was the most effective social media tool, with the highest number of followers and greatest engagement, mainly for females, a finding consistent with other research [28]. Videos of the two TV ads and a syphilis animation were the most popular content published on the campaign’s Facebook page. This finding suggests sensitive sexual health information is more palatable to young people when presented in video format. Past research has, however, revealed that the Facebook algorithm favours video posts above other types of posts [29]. Further research exploring post sentiment to determine the content that resonates most with the target audience is warranted.

The lack of similar programs with comparable data precludes inferences about the level of engagement on Facebook for this campaign. However, gender may moderate engagement with sexual health content on social media. Australian research suggests that young females are more responsive than young males to text messages containing sexual health information and are more likely to seek STI testing or talk to a doctor about their sexual health following receipt of such messages [30]. Exploring the cultural compatibility of this for Aboriginal young people is recommended.

Facebook was a preferred information channel for young people. High levels of engagement with Facebook have been previously reported amongst Aboriginal young people [31]. However, use does not equate to engagement for all topics [32], particularly stigmatised topics such as sexual health. This highlights the complexity of using social media for health promotion interventions; the user decides the content they will give due attention (view, read, and/or share) and the content they will ignore [33]. Research on social media use among young people, including Aboriginal young people, suggests a vital role in identity formation and the development of interpersonal relationships [34, 35]. Consequently, some young people may not engage with the sexual health content on these platforms due to fear of stigma or being bullied, or to preserve their peer and social identity [34, 35]. Using humour to encourage young people to engage with the sexual health content on social media may help defuse related shame [34]. Health messages communicated via social media have demonstrated greater reach and impact when key characteristics, such as emotion (e.g., personal stories) and practical value (e.g., educational messages), are incorporated [36].

### Limitations

Findings should be considered in conjunction with the limitations of a single time point (post only), cross-sectional evaluation design. Logistics and funding limitations prevented face to face data collection, which may have otherwise yielded more contextual and localised data on factors influencing campaign success for different communities. The possible influences of response bias associated with the largely female participant groups, along with social desirability and recall bias are also acknowledged.

Our sample size was small, precluding: 1) detection of significant differences within the two groups; and 2) comparison of survey findings by age group, gender, and region. Other research reports similar challenges recruiting Aboriginal people in research [37, 38] attributed to several factors, including past unethical research practices and participant burnout [39]. Adopting a decolonising approach to Aboriginal health research, including utilising peer researchers, may enhance future research participation [40]. Smaller participant numbers can be characteristic of research involving Aboriginal people from remote communities [41], and while some may challenge the integrity of the data, others will argue that data collected in this context are highly valued, making a meaningful contribution to the limited evidence base [42]. With the lack of research reporting on youth sexual health in remote Australian contexts, this evaluation responds to the overwhelming need to shape positive sexual health narratives and provide a platform for young Aboriginal people’s voices.

It was impossible to isolate outcomes specific to the *YDSF* campaign intervention [16]. With syphilis endemic in some Aboriginal communities, there are efforts external to the campaign to increase testing for syphilis-affected communities. Similarly, the broader *Young Deadly Free* (*YDF*) health promotion project may have contributed to the campaign outcomes.

### Implications

The *Young Deadly Syphilis Free* campaign evaluation findings show great promise. At no other point in time has a multi-media sexual health campaign been trialled across several jurisdictions in Australia addressing such a sensitive issue. *YDSF* challenged cultural protocols that typically preclude sexual health discussions in mixed gender settings and tested the palatability of such a campaign with the broader community. The positive engagement of young people with the topic during formative research and via social media, along with the evaluation findings, suggest it may be acceptable to publicly promote STI awareness among Aboriginal young people across multiple jurisdictions. Rigorous testing is however recommended with a diverse sample of the target group.

Optimising longer-term campaign impacts requires sustained effort and consideration of the diversity among Aboriginal people from different regions. In recognition of this, the *YDSF* campaign remains active with the support of further Commonwealth and State funding. New resources and ads featuring local community members from a range of jurisdictions have been developed in response to the feedback provided by the primary and secondary target audiences to increase campaign engagement. Embedding *YDSF* as part of a broader socioecological approach to address the range of determinants influencing sexual health outcomes, including universal strategies to address shame and stigma, will also help to change the narrative around sexual health and encourage positive behaviour change.

## Conclusion

The *Young Deadly Syphilis Free* campaign evaluation findings present a valuable case study of the impact of a mass media campaign on awareness, knowledge, attitudes, and behavioural intentions in Aboriginal youth sexual health promotion. Findings of this evaluation highlight the value of formative research coupled with the use of humour, a strengths-based approach (avoiding anatomical language and stigmatising Aboriginal people), and delivery via a mix of media channels (TV, Facebook, website) appear to be important factors in amplifying awareness of key sexual health messages and positively influencing future behaviours of young Aboriginal people aged 15–34 years and health and community workers across multiple regions. The study contributes to the modest literature, which reports on the design and delivery of mass media campaigns in Aboriginal sexual health, by offering insights into how to communicate with Aboriginal young people about sensitive sexual health issues across large geographical tracts and across diverse communities.

## Supporting information

S1 File Young people survey. (DOCX)

S2 File Health and community worker survey. (DOCX)
