# Supplementary material for: Evaluation of the Young, Deadly, Syphilis Free multi-media campaign in remote Australia
Source: PLoS One. 2022 Sep 9;17(9):e0273658. doi: 10.1371/journal.pone.0273658 (PMC9462794; doi:10.1371/journal.pone.0273658)
Supplement: S1 File — (DOCX) [file pone.0273658.s001.docx]

Young, Deadly, Syphilis Free Evaluation Survey for Young People

Start of Block: Block 1 - Consent

| 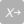 |
| --- |

Q1 Thank you for visiting the Young, Deadly, Free website.
This survey is about health advertisements you may have seen or heard on TV or radio.
The survey asks what you think of the ads so we can decide whether ads like these are useful for informing Aboriginal and Torres Strait Islander communities about public health issues.
There are no right or wrong answers to the questions. You can stop the survey at any time. Your answers to the questions cannot be linked to you.
Click on the 'Yes' box below if you would like to do the survey. It should take about 5 minutes.
If you don't want to do the survey, just click 'No'. That's fine - nothing bad will happen to you.  

- Yes, I would like to take part in the survey (1)
- No. **Thank you for your time.** (2)

End of Block: Block 1 - Consent

Start of Block: Block 2 - Screening 1

| 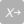 |
| --- |

Q2 A little about you ....
I am Aboriginal and/or Torres Strait Islander

- Yes (1)
- No. **Thank you for your time.** (2)

End of Block: Block 2 - Screening 1

Start of Block: Block 3 - Screening 2

Q3 How old are you?
Please select your age (years) from dropdown list

▼ 14 or younger (1) ... 35 or older (22)

End of Block: Block 3 - Screening 2

Start of Block: Block 4 - Screening 3

| 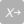 |
| --- |

Q4 Where do you live?

- South Australia (1)
- Western Australia (2)
- Northern Territory (3)
- Queensland (4)
- I live somewhere else in Australia (5)

End of Block: Block 4 - Screening 3

Start of Block: Block 5 - Demographics

| 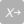 |
| --- |

Q5 Is English your first language?

- Yes (1)
- No (2)

| Page Break |  |
| --- | --- |

| 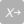 |
| --- |

Q6 Are you?

- Male (1)
- Female (2)
- Other (please specify) (3) ________________________________________________

| Page Break |  |
| --- | --- |

| 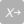 |
| --- |

Q7 What is the highest level of education you have completed?

- I completed primary school only (1)
- I left school before finishing Year 10 (2)
- I completed Year 10 (3)
- I completed Year 12 (4)
- I completed a diploma or university degree (5)

| Page Break |  |
| --- | --- |

| 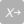 |
| --- |

Q8 Overall, how would you rate your health during the past 4 weeks?

- Excellent (1)
- Good (2)
- Fair (3)
- Poor (4)

| Page Break |  |
| --- | --- |

End of Block: Block 5 - Demographics

Start of Block: Block 6 - Survey

| 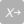 |
| --- |

Q9 Have you seen or heard any advertisements about health issues recently?

- Yes (1)
- No (2)

Skip To: Q11 If Have you seen or heard any advertisements about health issues recently? = No

Q10 Can you please describe the advertising you saw?

________________________________________________________________

________________________________________________________________

________________________________________________________________

________________________________________________________________

________________________________________________________________

| 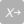 |
| --- |

Q11 Have you seen or heard any advertisements about syphilis recently?

- Yes (1)
- No (2)

Skip To: Q14 If Have you seen or heard any advertisements about syphilis recently? = No

Q12 Can you please describe the advertising you saw?

________________________________________________________________

________________________________________________________________

________________________________________________________________

________________________________________________________________

________________________________________________________________

| 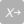 |
| --- |

Q13 Where did you see or hear this?
Tick as many boxes as needed

- Television (1)
- Facebook (2)
- Instagram (3)
- Diva Chat (4)
- Twitter (5)
- Radio (6)
- Young, Deadly, Free website (7)
- Somewhere else (please specify) (8) ________________________________________________

| 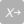 |
| --- |

Q14 There has recently been a campaign running in our communities called the Young, Deadly, Syphilis Free campaign. Here are some images from two TV advertisements from this campaign.
 Advertisement 1    
Advertisement 2

    

Before today, had you seen these ads?

- Yes, I've seen both ads (1)
- Yes, I've seen ad 1 (2)
- Yes, I've seen ad 2 (3)
- No, I haven't seen either ad (4)
- Don't know / unsure (5)

Skip To: Q20 If There has recently been a campaign running in our communities called the Young, Deadly, Syphilis Fre = No, I haven't seen either ad

Skip To: Q20 If There has recently been a campaign running in our communities called the Young, Deadly, Syphilis Fre = Don't know / unsure

| 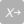 |
| --- |

Q15 Do you remember these messages in the ads?

|  | Yes (1) | No (2) | Don't know (3) | This was not a message in the ads (4) |
| --- | --- | --- | --- | --- |
| Syphilis is an infection that is spreading through our communities (1) |  |  |  |  |
| You can get syphilis if you have sex without a condom (2) |  |  |  |  |
| Syphilis can harm you and the people you sleep with too (3) |  |  |  |  |
| Syphilis can harm unborn babies (4) |  |  |  |  |
| Pregnant women should get tested for syphilis (5) |  |  |  |  |
| Young people should use condoms and get tested for syphilis (6) |  |  |  |  |
| Syphilis can be easily treated with antibiotics (7) |  |  |  |  |

| 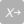 |
| --- |

Q16 Please tell us whether you agree or disagree with these statements about the ads

|  | Agree (1) | Disagree (2) | Don't know / unsure (3) |
| --- | --- | --- | --- |
| I enjoyed watching these ads (1) |  |  |  |
| These ads told me something new (2) |  |  |  |
| These ads are relevant to me (3) |  |  |  |
| These ads are believable (4) |  |  |  |
| I would talk about these ads with my friends (5) |  |  |  |
| These ads were easy to understand (6) |  |  |  |
| These ads grabbed my attention (7) |  |  |  |

| 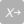 |
| --- |

Q17 When you saw or heard the TV ads, did they make you think about doing anything differently?

- Yes (1)
- No (2)
- Don't know / unsure (3)

Skip To: Q19 If When you saw or heard the TV ads, did they make you think about doing anything differently? = No

Skip To: Q19 If When you saw or heard the TV ads, did they make you think about doing anything differently? = Don't know / unsure

| 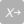 |
| --- |

Q18 What did you think about doing differently?
Tick as many boxes as needed

- Get tested for syphilis (1)
- Use condoms when having sex (2)
- Talk to family and friends about the importance of getting tested for syphilis (3)
- Other (please specify) (4) ________________________________________________

| 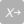 |
| --- |

Q19 Using a scale of 1 (disagree) to 5 (agree), how much do you agree with the below statements?

|  | 1 (Disagree) (1) | 2 (2) | 3 (3) | 4 (4) | 5 (Agree) (5) |
| --- | --- | --- | --- | --- | --- |
| These ads have changed the way people in the community think about syphilis (1) |  |  |  |  |  |
| These ads have increased knowledge about syphilis in the community (2) |  |  |  |  |  |
| These ads have helped people in the community understand how to stay syphilis free (3) |  |  |  |  |  |
| These ads have helped to reduce the shame and stigma about syphilis and/or sexually transmissible infections (4) |  |  |  |  |  |
| These ads have helped to reduce the shame and stigma about getting tested for syphilis and/or sexually transmissible infections (5) |  |  |  |  |  |
| Young people feel more confident to get tested regularly for syphilis and/or sexually transmissible infections because of these ads (6) |  |  |  |  |  |

| 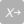 |
| --- |

Q20 Please indicate how long you spend reading, listening, or using the below each day

|  | I don't use this (1) | Less than 1 hour (2) | 1 to 2 hours (3) | 2 to 3 hours (4) | 3 hours or more (5) |
| --- | --- | --- | --- | --- | --- |
| Television (1) |  |  |  |  |  |
| Radio (2) |  |  |  |  |  |
| Facebook (3) |  |  |  |  |  |
| Instagram (4) |  |  |  |  |  |
| Twitter (5) |  |  |  |  |  |
| Diva Chat (6) |  |  |  |  |  |
| Other (please specify) (7) |  |  |  |  |  |

| 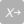 |
| --- |

Q21 Which is your favourite way to get information?

- Television (1)
- Radio (2)
- Facebook (3)
- Instagram (4)
- Twitter (5)
- Diva Chat (6)
- Other (please specify) (7) ________________________________________________

Q22 Thank you for completing this survey.   If you want to enter a draw to win an iPad, please provide your name and contact number or email address below.   Your contact details will not be linked to your survey responses.

________________________________________________________________

________________________________________________________________

________________________________________________________________

________________________________________________________________

________________________________________________________________

End of Block: Block 6 - Survey
