# Supplementary material for: Evaluation of the Young, Deadly, Syphilis Free multi-media campaign in remote Australia
Source: PLoS One. 2022 Sep 9;17(9):e0273658. doi: 10.1371/journal.pone.0273658 (PMC9462794; doi:10.1371/journal.pone.0273658)
Supplement: S2 File — (DOCX) [file pone.0273658.s002.docx]

Young, Deadly, Syphilis Free Evaluation Survey for Health and Community Workers

Start of Block: Block 1 - Consent

Q1 Thank you for visiting the Young, Deadly, Free website.
This survey is about health advertisements you may have seen or heard on TV or radio.
The survey asks what you think of the ads so we can decide whether ads like these are useful for informing people in Aboriginal and Torres Strait Islander communities about public health issues.
There are no right or wrong answers to the questions. You can stop the survey at any time. Your answers to the questions cannot be linked to you.
Click on the 'Yes' box below if you would like to do the survey. It should take about 5 minutes.
If you don't want to do the survey, just click 'No'.

- Yes, I would like to take part in the survey. (1)
- No. **Thank you for your time.** (2)

End of Block: Block 1 - Consent

Start of Block: Block 2 - Screening

| 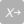 |
| --- |

Q2 Which state or territory do you work in?

- South Australia (1)
- Western Australia (2)
- Northern Territory (3)
- Queensland (4)
- I work elsewhere in Australia (5)

End of Block: Block 2 - Screening

Start of Block: Block 3 - Demographics

| 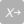 |
| --- |

Q3 I am

- Male (1)
- Female (2)
- Other (3)

| 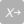 |
| --- |

Q4 What is your current age?

▼ 18 - 24 years (1) ... 75 years or older (7)

| 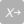 |
| --- |

Q5 What is your country of birth?

- Australia (1)
- Other (please specify) (2) ________________________________________________

Display This Question:

If What is your country of birth? = Other (please specify)

| 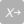 |
| --- |

Q6 How long have you lived in Australia?

▼ Less than 12 months (1) ... 21 years or more (6)

| 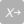 |
| --- |

Q7 Do you identify as being Aboriginal and/or Torres Strait Islander?

- Yes (1)
- No (2)

| 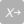 |
| --- |

Q8 Is English your first language?

- Yes (1)
- No (please specify main language) (2) ________________________________________________

Display This Question:

If Is English your first language? = No (please specify main language)

| 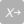 |
| --- |

Q9 How well do you speak English?

- Very well (1)
- Well (2)
- Not well (3)

| 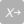 |
| --- |

Q10 What is the highest level of education you have completed?

- Primary School (1)
- Year 10 (2)
- Year 12 (3)
- Trade / Diploma certificate (4)
- University / College (5)
- Other (please specify) (6) ________________________________________________

| 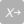 |
| --- |

Q11 Which of the following best describes your primary work?

- Health Worker (1)
- Nurse (2)
- Doctor (3)
- Youth Worker (4)
- Community Worker (5)
- Other (please specify) (6) ________________________________________________

End of Block: Block 3 - Demographics

Start of Block: Block 4 - Survey

| 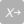 |
| --- |

Q12 Have you seen or heard any advertisements about health issues recently?

- Yes (1)
- No (2)

Skip To: Q14 If Have you seen or heard any advertisements about health issues recently? = No

Q13 Can you describe the advertising you saw?

________________________________________________________________

________________________________________________________________

________________________________________________________________

________________________________________________________________

________________________________________________________________

| 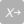 |
| --- |

Q14 Have you seen or heard any advertisements about syphilis recently?

- Yes (1)
- No (2)

Skip To: Q17 If Have you seen or heard any advertisements about syphilis recently? = No

Q15 Can you please describe the advertising you saw?

________________________________________________________________

________________________________________________________________

________________________________________________________________

________________________________________________________________

________________________________________________________________

| 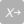 |
| --- |

Q16 Where did you see or hear this?
Tick as many boxes as needed.

- Television (1)
- Facebook (2)
- Instagram (3)
- Diva Chat (4)
- Twitter (5)
- Radio (6)
- Young, Deadly, Free website (7)
- Somewhere else (please specify) (8) ________________________________________________

| 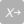 |
| --- |

Q17 There has recently been a campaign running in our communities called the Young, Deadly, Syphilis Free campaign. Here are some images from two TV advertisements from this campaign.
Advertisement 1
    
Advertisement 2

    

Before today, had you seen these ads?

- Yes, I've seen both ads (1)
- Yes, I've seen ad 1 (2)
- Yes, I've seen ad 2 (3)
- No, I haven't seen either ad (4)
- Don't know / unsure (5)

Skip To: Q22 If There has recently been a campaign running in our communities called the Young, Deadly, Syphilis Fre = No, I haven't seen either ad

Skip To: Q22 If There has recently been a campaign running in our communities called the Young, Deadly, Syphilis Fre = Don't know / unsure

| 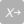 |
| --- |

Q18 Do you remember these messages in the ads?

|  | Yes (1) | No (2) | Don't know (3) | This was not a message in the ads (4) |
| --- | --- | --- | --- | --- |
| Syphilis is an infection that is spreading through our communities (1) |  |  |  |  |
| You can get syphilis if you have sex without a condom (2) |  |  |  |  |
| Syphilis can harm you and the people you sleep with too (3) |  |  |  |  |
| Syphilis can harm unborn babies (4) |  |  |  |  |
| Pregnant women should get tested for syphilis (5) |  |  |  |  |
| Young people should use condoms and get tested for syphilis (6) |  |  |  |  |
| Syphilis can be easily treated with antibiotics (7) |  |  |  |  |

| 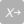 |
| --- |

Q19 Please tell us whether you agree or disagree with these statements about the ads.

|  | Agree (1) | Disagree (2) | Don't know / Unsure (3) |
| --- | --- | --- | --- |
| The ads are relevant to young people in my community (1) |  |  |  |
| The ads are believable (2) |  |  |  |
| The ads were easy to understand (3) |  |  |  |
| These ads would appeal to young people in my community (4) |  |  |  |
| I would talk about these ads with young people in my community (5) |  |  |  |

| 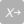 |
| --- |

Q20 After seeing or hearing the ads, what changes have you made (if any) to the way you practice?
Tick all boxes that apply.

- I offer more opportunistic testing to young people attending my health service (1)
- I talk more often to young people attending my health service about STIs and the importance of testing (2)
- I offer free condoms to all young people who attend my health service (3)
- I talk more often to young people in the community about STIs and the importance of testing (4)
- Other (please specify) (5) ________________________________________________
- I have not made any changes (6)

| 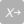 |
| --- |

Q21 Using a scale of 1 (disagree) to 5 (agree), how much do you agree with the below statements?

|  | 1 (Disagree) (1) | 2 (2) | 3 (3) | 4 (4) | 5 (Agree) (5) |
| --- | --- | --- | --- | --- | --- |
| These ads have changed the way people in the community think about syphilis (1) |  |  |  |  |  |
| These ads have increased knowledge about syphilis in the community (2) |  |  |  |  |  |
| These ads have helped people in the community understand how to stay syphilis free (3) |  |  |  |  |  |
| These ads have helped to reduce the shame and stigma about syphilis and/or sexually transmissible infections (4) |  |  |  |  |  |
| These ads have helped to reduce the shame and stigma about getting tested for syphilis and/or sexually transmissible infections (5) |  |  |  |  |  |
| Young people feel more confident to get tested regularly for syphilis and/or sexually transmissible infections because of these ads (6) |  |  |  |  |  |

| 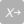 |
| --- |

Q22 Have you experienced more young people requesting syphilis testing since the campaign commenced in July 2017?

- Yes (1)
- No (2)
- This question is not relevant to the type of work I do (3)

Skip To: Q24 If Have you experienced more young people requesting syphilis testing since the campaign commenced i... = No

Skip To: Q24 If Have you experienced more young people requesting syphilis testing since the campaign commenced i... = This question is not relevant to the type of work I do

| 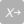 |
| --- |

Q23 What is your estimate of how many more requests for testing you have experienced since the start of the campaign?

- 10% more (1)
- 50% more (2)
- 80% more (3)
- 100% more (4)

Q24 Please provide any other comments you have about the syphilis campaign below.

________________________________________________________________

________________________________________________________________

________________________________________________________________

________________________________________________________________

________________________________________________________________

End of Block: Block 4 - Survey
